# Artificial intelligence, machine learning, and deep learning in rhinology: a systematic review

**DOI:** 10.1007/s00405-022-07701-3

**Published:** 2022-10-19

**Authors:** Antonio Mario Bulfamante, Francesco Ferella, Austin Michael Miller, Cecilia Rosso, Carlotta Pipolo, Emanuela Fuccillo, Giovanni Felisati, Alberto Maria Saibene

**Affiliations:** 1 Otolaryngology Unit, Santi Paolo e Carlo Hospital, Department of Health Sciences, Università degli Studi di Milano, Milan, Italy; 2 Ohio University Heritage College of Osteopathic Medicine, Dublin, OH USA; 3 Department of Clinical Sciences and Translational Medicine, Università degli Studi di Roma Tor Vergata, Rome, Italy

**Keywords:** Algorithm, Radiomics, Anatomy, Paranasal sinuses, Allergy, Rhinitis

## Abstract

**Purpose:**

This PRISMA-compliant systematic review aims to analyze the existing applications of artificial intelligence (AI), machine learning, and deep learning for rhinological purposes and compare works in terms of data pool size, AI systems, input and outputs, and model reliability.

**Methods:**

MEDLINE, Embase, Web of Science, Cochrane Library, and ClinicalTrials.gov databases. Search criteria were designed to include all studies published until December 2021 presenting or employing AI for rhinological applications. We selected all original studies specifying AI models reliability. After duplicate removal, abstract and full-text selection, and quality assessment, we reviewed eligible articles for data pool size, AI tools used, input and outputs, and model reliability.

**Results:**

Among 1378 unique citations, 39 studies were deemed eligible. Most studies (*n* = 29) were technical papers. Input included compiled data, verbal data, and 2D images, while outputs were in most cases dichotomous or selected among nominal classes. The most frequently employed AI tools were support vector machine for compiled data and convolutional neural network for 2D images. Model reliability was variable, but in most cases was reported to be between 80% and 100%.

**Conclusions:**

AI has vast potential in rhinology, but an inherent lack of accessible code sources does not allow for sharing results and advancing research without reconstructing models from scratch. While data pools do not necessarily represent a problem for model construction, presently available tools appear limited in allowing employment of raw clinical data, thus demanding immense interpretive work prior to the analytic process.

## Introduction

“The Skynet Funding Bill is passed. The system went online August 4th, 1997. Human decisions are removed from strategic defense. Skynet begins to learn at a geometric rate. It becomes self-aware at 2:14 a.m. Eastern time, August 29th” [1]. Introducing the second chapter of the Terminator franchise, director James Cameron insinuates a gritty, yet precise, definition of what is usually defined as general artificial intelligence (AI), i.e., a machine perfectly mimicking human intelligence. Outside of science fiction and speculation, we are limited to working with narrow AI: electronic systems created with the capacity to substitute for humans in various specific tasks. When integrated with machine learning (ML) algorithms, an AI is allowed to learn and improve from experience, becoming progressively capable to learn how to execute specific tasks even if it has not been specifically programmed to do it ab initio. However, ML algorithms still require human intervention in the training phase. More recently introduced deep learning (DL) models are specific ML applications whose complex algorithms and neural nets (consisting of many hierarchical layers—i.e., deep—of non-linear processing units) train models, with little to no explicit human data input. These progressive developments make AI an incredible tool in various fields, including healthcare, where it has been deemed suitable for repetitive analytic tasks [2], complex calculations [3], and complex forecasts [4, 5].

Rhinology is not immune to such tasks considering procedures, such as nasal cytology smears analysis (repetitive task), nasal airflow computational fluid dynamics modeling (complex calculations), and radiomics-based oncological risk stratification (complex forecast).

Several intrinsic technical issues make AI applications in rhinology challenging and embryonic at best. First, researchers must choose from several computational techniques for ML, many of which have been used in different situations based on complex program decision-making [6]. Different computational techniques require different input data (qualitative and quantitative) for algorithm training, results validation, and so-called “truth” imposition on the AI. Furthermore, commonly used clinical data, particularly those in graphical forms, such as radiologic studies or histology slides, require heavy manipulation before being fed to the AI. Finally, currently available rhinological AI studies rely on different algorithms developed de novo for nearly every study rather than sharing open-access infrastructures facilitating progressive development.

This systematic review aims at analyzing the existing literature on AI applications in rhinology, defining technologies, data sets, and inputs appropriate for AI/ML/DL, verifying the real-world verified applications, and determining whether AI in rhinology might benefit from a stricter commitment to open science.

## Methods

### Search strategy

After PROSPERO database registration (ID CRD42022298020), a systematic review was conducted between December 15, 2021, and April 30, 2022, according to the PRISMA reporting guidelines [7]. We conducted systematic electronic searches for studies in the English, Italian, German, French and Spanish languages reporting original data concerning AI, ML, or DL applications in human rhinology.

On December 15, 2021, we searched the MEDLINE, Embase, Web of Science, Cochrane Library, and ClinicalTrials.gov databases for AI-related terms in association with rhinology-, nose- or paranasal sinuses-related terms. Full search strategies and the number of items retrieved from each database are available in Table 1.

**Table 1:** Search strategy details and items retrieved from each consulted database

Database	Search query	Items retrieved
Medline	(“artificial intelligence”[All Fields] OR “machine learning”[All Fields] OR “knowledge engineering”[All Fields] OR “deep learning”[All Fields]) AND (“nose”[MeSH Terms] OR “nose”[All Fields] OR “paranasal sinus”[All Fields] OR “maxillary sinus”[All Fields] OR “ethmoid sinus”[All Fields] OR “sphenoid sinus”[All Fields] OR “frontal sinus”[All Fields] OR “nasal cavity”[All Fields] OR (“rhinology”[Journal] OR “rhinol suppl”[Journal] OR “rhinology”[All Fields]))	311
Web of Science	(“artificial intelligence” OR “machine learning” OR “knowledge engineering” OR “deep learning”) AND (nose OR “paranasal sinus” OR “maxillary sinus” OR “ethmoid sinus” OR “sphenoid sinus” OR “frontal sinus” OR “nasal cavity” OR rhinology)	631
Embase	(‘artificial intelligence’ OR ‘machine learning’ OR ‘knowledge engineering’ OR ‘deep learning’) AND (‘nose’ OR ‘paranasal sinus’ OR ‘maxillary sinus’ OR ‘ethmoid sinus’ OR ‘sphenoid sinus’ OR ‘frontal sinus’ OR ‘nasal cavity’ OR ‘rhinology’)	444
Scopus	TITLE-ABS-KEY ((“artificial intelligence” OR “machine learning” OR “knowledge engineering” OR “deep learning”) AND (nose OR “paranasal sinus” OR “maxillary sinus” OR “ethmoid sinus” OR “sphenoid sinus” OR “frontal sinus” OR “nasal cavity” OR rhinology))	1038
Cochrane Library	(“artificial intelligence” OR “machine learning” OR “knowledge engineering” OR “deep learning”) AND (nose OR “paranasal sinus” OR “maxillary sinus” OR “ethmoid sinus” OR “sphenoid sinus” OR “frontal sinus” OR “nasal cavity” OR rhinology) in Title Abstract Keyword—(Word variations have been searched)	7

We included articles, where AI, ML, or DL was explicitly used by the authors for any rhinological purpose in humans providing model reliability metrics. We excluded meta-analyses and systematic and narrative reviews, which were nevertheless hand-checked for additional potentially relevant studies. No minimum study population was required.

Abstracts and full texts were reviewed in duplicate by different authors. At the abstract review stage, we included all studies deemed eligible by at least one rater. At the full-text review stage, disagreements were resolved by consensus between raters.

### PICOS criteria

The PICOS (Population, Intervention, Comparison, Outcomes, and Study) framework [7] for the review was:

P: any patient with confirmed or potential rhinological conditions or simply acting as a model of sinonasal anatomy or rhinological conditions.

I: any application of artificial intelligence for rhinological diagnostic, therapeutic, classification, or speculative purposes.

C: no comparator available.

O: effectiveness of created models.

S: all original study types.

For each article, we recorded: country of origin, type of article (whether technical or clinical and indicating the study type for the latter group), data set numerosity with train:validation:test split ratios, type of input, type of output, type of AI model, broad field of application, specific model application, model reliability, and source code availability. Data extraction was performed in duplicate by different authors (AMB and AMS) and disagreements were solved by consensus.

Clinical studies were assessed for both quality and methodological bias according to the National Heart, Lung, and Blood Institute Study Quality Assessment Tools (NHI-SQAT) [8]. Articles were rated in duplicate by two authors and disagreements were resolved by consensus. Items were rated as good if they fulfilled at least 80% of the items required by the NHI-SQAT, fair if they fulfilled between 50% and 80% of the items, and poor if they fulfilled less than 50% of the items, respectively.

The level of evidence for clinical studies was scored according to the Oxford Centre for evidence-based medicine (OCEBM) level of evidence guide [9].

Due to the significant heterogeneity of study populations and methods and the predominantly qualitative nature of collected data, no meta-analysis was originally planned or performed a posteriori.

## Results

**Fig. 1:**
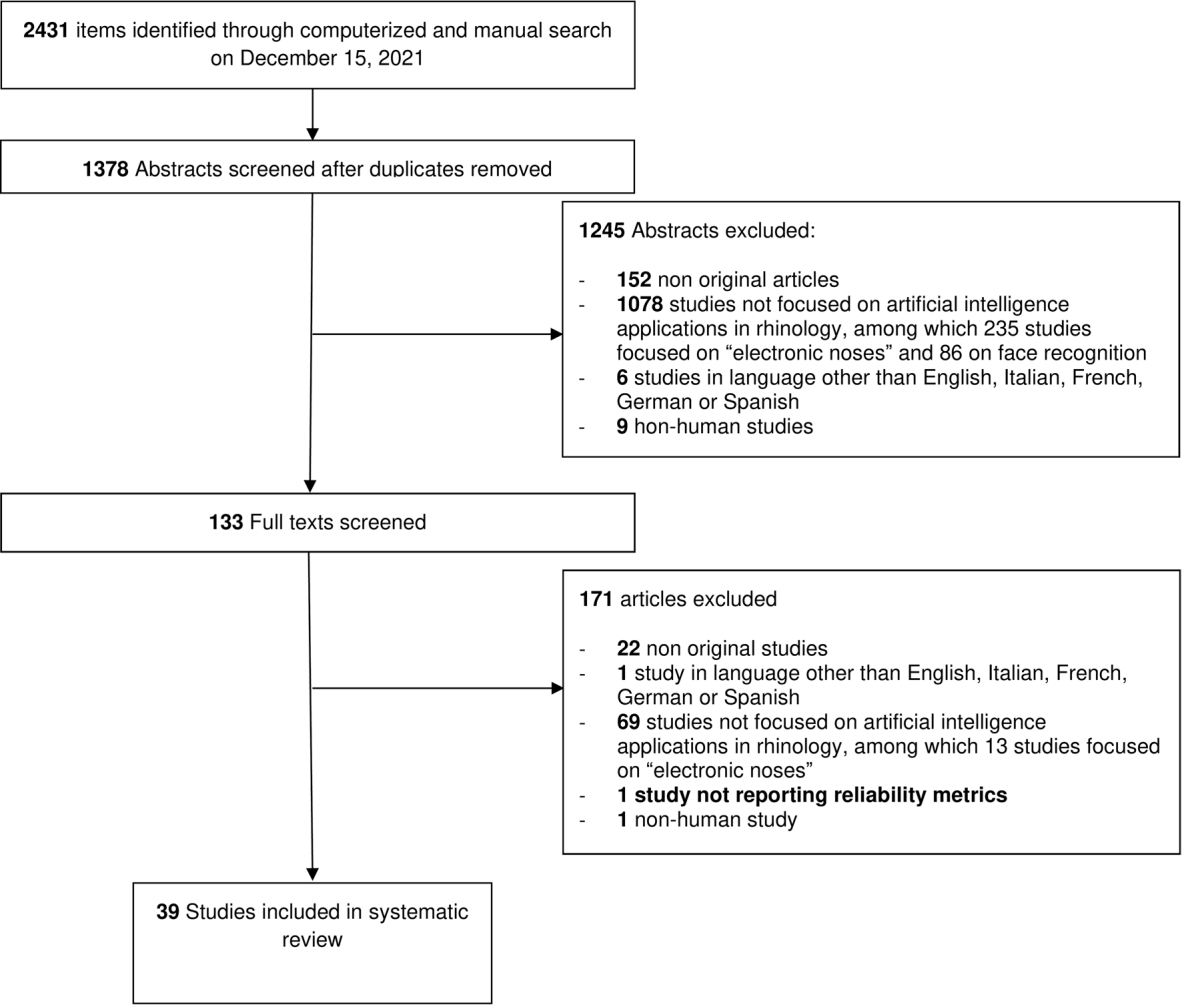
PRISMA-style flow diagram of study selection during the systematic review process

Among the 1378 unique research items initially identified, a total of 133 articles were selected for full-text evaluation. No further study was identified for full-text evaluation after reference checking. Thirty-nine studies published between 1997 and 2021 were retained for analysis (see Fig. 1) [2–4, 6, 10–44]. Most studies were published in the last 5 years. Eleven of these studies were completed in the United States (US), with South Korea being the second most productive country (*n* = 5). Publications were collected from 14 different countries on four continents. Twenty-nine studies were purely technical in their structure. The remaining 10 clinical articles were retrospective cohort studies (*n* = 3), prospective cohort studies (*n* = 6), and a single case series. Accordingly, their level of evidence according to the OCEBM scale was IV (*n* = 1), III (*n* = 3), and II (*n* = 2). Clinical articles were rated as good (*n* = 7) or fair (*n* = 3) according to the NHI-SQAT tools, with no article being rated as low quality. No significant biases toward the objectives of our systematic review were identified. Table 2 reports the country of origin, evidence, and quality rating (where available) for all studies.

**Table 2:** Country of origin, and evidence and quality rating of reviewed articles

Authors	Country of origin	Type of paper	OCEBM rating	NHI-SQAT rating
Aggelides et al., 2020 [10]	Greece	Technical	N/A	N/A
Arfiani et al., 2019 [6]	Indonesia	Technical	N/A	N/A
Bieck et al., 2020 [11]	Germany	Technical	N/A	N/A
Borsting et al., 2020 [12]	US	Technical	N/A	N/A
Chowdhury et al., 2020 [13]	US	Clinical (PCS)	II	good
Chowdhury et al., 2019 [14]	US	Clinical (PCS)	II	Good
Hasid et al., 1997 [15]	Belgium	Clinical (PCS)	II	Fair
Dimauro et al., 2019 [16]	Italy	Technical	N/A	N/A
Dimauro et al., 2020a [17]	Italy	Technical	N/A	N/A
Dimauro et al., 2020b [18]	Italy	Technical	N/A	N/A
Dorfman et al., 2020 [19]	US	Clinical (CS)	IV	Good
Kannan et al., 2020 [20]	India	Technical	N/A	N/A
Farhidzadeh et al., 2016 [21]	US	Technical	N/A	N/A
Fujima et al., 2019 [22]	Japan	Technical	N/A	N/A
Girdler et al., 2021 [23]	US/South Korea	Technical	N/A	N/A
Huang et al., 2020 [24]	Australia	Technical	N/A	N/A
Humphries et al., 2020 [25]	US	Clinical (RCS)	III	Good
Jeon et al., 2021 [26]	South Korea	Technical	N/A	N/A
Jung et al., 2021 [27]	South Korea	Technical	N/A	N/A
Kim et al., 2019a [28]	South Korea	Technical	N/A	N/A
Kim et al., 2019b [4]	South Korea	Technical	N/A	N/A
Kim et al., 2021 [2]	South Korea	Clinical (PCS)	II	Fair
Kuwana et al., 2021 [29]	Japan	Technical	N/A	N/A
Lamassoure et al., 2021 [3]	France	Technical	N/A	N/A
Laura et al., 2019 [30]	Germany	Technical	N/A	N/A
Lötsch et al., 2021 [31]	Germany	Clinical (PCS)	II	Good
Murata et al., 2019 [32]	Japan	Technical	N/A	N/A
Neves et al., 2021 [33]	US	Technical	N/A	N/A
Parmar et al., 2020 [34]	Australia	Technical	N/A	N/A
Parsel et al., 2021 [35]	US	clinical (PCS)	II	good
Putri et al., 2021 [36]	Indonesia	Technical	N/A	N/A
Quinn et al., 2015 [37]	US	Technical	N/A	N/A
Ramkumar et al., 2017 [38]	US	Technical	N/A	N/A
Soloviev et al., 2020 [39]	Russia	Technical	N/A	N/A
Staartjes et al., 2021 [40]	Switzerland	Technical	N/A	N/A
Thorwarth et al., 2021 [41]	US	Clinical (RCS)	III	good
Wirasati et al., 2020 [42]	Indonesia	Technical	N/A	N/A
Wu et al., 2020 [43]	China	Technical	N/A	N/A
Wu et al., 2021 [44]	China	Clinical (RCS)	III	fair

*PCS* prospective cohort study, *RCS* retrospective cohort study, *OCEBM* Oxford centre for evidence-based medicine, *N/A* not available, *NHI-SQAT* National Heart, Lung, and Blood Institute Study Quality Assessment Tool

Data set numerosity order of magnitude ranged from 10^1^ to 10^4^*.* Train:validation:test splits (reported for 29 articles) were extremely varied. Four articles used variable train:validation:test splits. With one exception, train data sets outweighed test data sets, with ratios ranging from 2:1 to 30:1. There were multiple inputs including: manually compiled binary, continuous and/or categorical variables (*n* = 15), pre-elaborated bidimensional graphics (*n* = 12), native bidimensional graphics (*n* = 8), native tridimensional graphics (*n* = 3), and verbal fragments (*n* = 1). Most outputs (*n* = 23) were binary classifications of items processed from the AI (e.g., presence or absence of maxillary inflammation on a radiologic image), 9 were categorical classifications of items, and 7 were continuous estimates, such as cell counts and radiological volumes segmentations.

Regarding AI models and architectures employed, a convolutional neural network (CNN) was the most frequently used, particularly for graphic input elaboration, while support vector machine (SVM) was the most prevalent model for compiled data analysis. Eight articles purposely employed different AI models, often comparing them in terms of reliability. Sinonasal anatomy (*n* = 8), rhinosinusitis (*n* = 24), and allergy (*n* = 7) were the most frequent broad fields of application of AI models. These were also applied to endoscopic sinus surgery, sinonasal neoplasms, and rhinoplasty, although in fewer instances.

Specific AI applications were protean and extremely well-defined. Anatomical structure identification and segmentation, in addition to disease diagnosis from radiologic studies, represented the most frequent scenarios. Authors chose different metrics for AI model reliability, with accuracy (32–100%) and area under the curve (0.6–0.974) being the most frequently employed. AI software code availability was scarce. No code was available for two studies, six were built on a third-party open-source framework, three used precompiled free software (usually R), three used commercial software, and three others provided links to the code employed or formally stated free availability of such code upon request. Table 3 reports specific information on the AI models presented in the studies.

**Table 3:** Technical and methodological details of AI models presented in the reviewed studies

Authors	Data set numerosity	Training/validation/testing split	Type of input	Type of output	Type of AI model	AI technology employed	Brief description of applications	Reliability of model	Code availability
Aggelides et al., 2020 [10]	662 gestures	Variable T:T splits	COM (data from wristband device with accelerometer and gyroscope)	CAT (interpretation of gesture type according to own classification)	4 KNN, SVM, RF, DT	DL	Recognizing gestures associated with allergic rhinitis	ACC 0.93	No
Arfiani et al., 2019 [6]	200 CT scans	Variable T:T splits	COM (patient data and maxillary sinus Hounsfield unit)	BIN (diagnosis of acute sinusitis)	KSPKM, SVM	ML	Diagnosing acute sinusitis from data compiled from CT scans	ACC 0.97 (KSPKM), 0.90 (SVM)	No
Bieck et al., 2020 [11]	3850 navigation workflow sentences	9:1T:T split	VER (descriptions of the endoscope state)	CAT (prediction of next action and landmark in ESS)	S2S, TRF, HMM, LSTM	ML	Predicting next steps or landmarks in ESS from surgical annotations	ACC 0.53 (TRF), 0.35 (LSTM), 0.32 (S2S), 0.83 at sentence-level	No
Borsting et al., 2020 [12]	18,148 pre- and post-rhinoplasty images	8:1:1T:V:T split	N2D (pre- and post-rhinoplasty photos)	BIN (identification of patients who underwent rhinoplasty)	CNN	DL	Recognizing patients who underwent rhinoplasty	CCR on true rhinoplasty status 0.67 (95% CI 0.59, 0.74)	Yes
Chowdhury et al., 2020 [13]	147 patients	n/a	COM (biochemical data, SNOT-22 and polyp status from single patients)	CON (reduction of SNOT-22 after surgery)	RF, DT	ML	Selecting main factors for SNOT-22 reduction after ESS	Mean squared errors are reported for each variable	Freeware built-in feature (R)
Chowdhury et al., 2019 [14]	Pre- and post-treatment single slides from CT scans from 239 patients	8:1:1T:V:T split	N2D (single images from head CT scan)	BIN (open or closed ostiomeatal complex)	CNN	DL	Recognizing ostiomeatal complex patency from CT scans	AUC 0.87 (95% CI 0.78–0.92)	Open source framework available
Hasid et al., 1997 [15]	49 patients	n/a	COM (clinical data, morphonuclear features, glycohistochemical features)	CAT (polyps classification according to own classification)	DT	ML	Evaluating features characterizing each class of polyps	ACC 0.959 (95% CI 0.86–0.995)	Open source framework available
Dimauro et al., 2019 [16]	4429 NCS cells	3,4:1T:T split	E2D (single cells from NCS)	BIN (recognition of cells), CAT (identification of cell type)	CNN	DL	Identifying and classifying cells in NCS	RV 0.99 on the test set and 0.94 on the validation set for cell categorization, 0.977 for single cells identification	No
Dimauro et al., 2020a [17]	326 NCS fields tiles	2.3:1T:T split	E2D (tiles from NCS)	BIN (identification of biofilm-producing bacterial colonies)	CNN	DL	Identifying biofilm-producing bacterial colonies	ACC 0.98	No
Dimauro et al., 2020b [18]	87 cytology fields (cells identification); 1990 cells (cells classification)	n/a (pre-trained model see art. 100)	N2D (fields from cytological centrifugation) and non-native 2d graphical (single cells)	BIN (recognition of cells), CAT (identification of cell type)	CNN	DL	Identifying and classifying cells in NCS	RV 0.523 (cell identification), 0.00–0.90 (cell classification, different for each cell type)	No
Dorfman et al., 2020 [19]	100 patients (pre- and post-operative images)	n/a (pre-trained commercial algorithm)	N2D (pre- and post-operative photos from open rhinoplasty patients)	CON (estimated patient age)	CNN	DL	Estimating patients age	Correlation coefficient with real age *r* = 0.91 on pre-operatory images	Commercial code
Kannan et al., 2020 [20]	872 patients	8:2T:T	COM (92 items from clinical data and allergy test results)	BIN (diagnosis of allergic rhinitis)	Genetic algorithm for selecting features and extreme learning machine for classification purposes	DL	Recognizing allergic rhinitis patients starting from allergy tests and clinical data	ACC 0.977	No
Farhidzadeh et al., 2016 [21]	25 nasopharyngeal carcinoma MRIs	n/a	E2D (contoured neoplasms from contrast-enhanced T1 MR images)	BIN (disease progression estimate)	SVM	ML	Estimating disease progression from radiomics features	ACC 0.76–0.80 AUC0.6–0.76	No
Fujima et al., 2019 [22]	36 sinonasal squamous cells carcinoma MRIs	8:1T:V	E2D (contoured neoplasms from MR images, with different parameters)	BIN (disease control estimate)	SVM	DL	Predicting local disease control	ACC 0.92, SEN 1, SPE 0.82, PPV 0.86, NPV 1	No
Girdler et al., 2021 [23]	222 endoscopy frames	15:3:2T:V:T	N2D (frames from endoscopy in normal patients, nasal polyps or inverted papilloma)	CAT (normal endoscopy, inverted papilloma or nasal polyp)	CNN	DL	Distinguishing normal endoscopy, nasal polyp and inverted papilloma images	ACC 0.742 ± .058	Yes
Huang et al., 2020 [24]	1063 single images from CT scans	3.4:1T:T	E2D (cropped single coronal CT slice at the anterior ethmoidal foramen)	BIN (anterior ethmoidal artery adherent or suspended in mesentery	CNN	DL	Identifying suspended anterior ethmoid arteries	ACC 0.827 (95% CI 0.777–0.878)	Open source framework available
Humphries et al., 2020 [25]	700 CT scans	14:4:51T:V:T	NVI (whole head CT scan)	CON (percentage of sinus opacification)	CNN	DL	Quantifying overall sinus opacification in CT scans	DSC mean 0.93; range 0.86–0.97	Open source framework available
Jeon et al., 2021 [26]	1535 patients	10:1:1T:V:T	N2D (Waters' and Caldwell's projection radiographs)	BIN (presence of maxillary, ethmoidal, or frontal sinusitis)	CCN on two networks	DL	Diagnosing sinusitis from Waters- or Caldwell projections	AUC 0.71 (95% CI 0.62–0.80) for Waters' view and 0.78 (95% CI 0.84–0.92) for Caldwell's view	No
Jung et al., 2021 [27]	123 cone beam CT scans	4:1:1T:V:T	NVI (whole head cone beam CT)	CON (maxillary sinus volume and air/lesion ratio inside)	CNN	DL	Identifying, segmenting, and defining in air/lesion level the maxillary sinus	Best DSC for air 0.93 ± .16, best DSC for lesions 0.77 ± 0.18	No
Kim et al., 2019a [28]	9340 radiographs	80:10:3.4T:V:T	E2D (maxillary sinus images from Waters' view radiographs)	BIN (diagnose maxillary sinusitis)	CNN	DL	Diagnosing maxillary sinusitis from Waters' radiograph	AUC 0.93 and 0.88 for the two different test sets	No
Kim et al., 2019b [4]	5020 radiographs	30:1T:T	E2D (trimmed Waters' view radiographs)	BIN (identify the maxillary sinus and diagnose maxillary sinusitis)	Majority decision algorithm on 3 CNN models	DL	Identifying the maxillary sinus and diagnosing maxillary sinusitis	ACC 0.941 and 0.9412, AUC 0.948 and 0.942 for internal and external test sets, respectively	No
Kim et al., 2021 [2]	129 patients	N/A	COM (clinical and histology features)	BIN (satisfactory surgical outcome)	DT and RF	ML	Predicting surgery outcomes from patient- and histology-specific variables	ACC 0.8404	Free add-on for free software
Kuwana et al., 2021 [29]	1168 ortopantomographs	3:1T:T	E2D (labeled images from orthopantomograph)	BIN (maxillary sinusitis diagnosis, presence of maxillary sinus cysts)	DetectNet neural network	DL	Locating the maxillary sinus on ortopantomographs and identifying healthy sinus, sinusitis and cysts	ACC 0.9–0.91, SEN 0.88–0.85 SPE 0.91–0.96 for maxillary sinusitis; ACC 0.97–1, SEN 0.8–1 SPE 1–1 for maxillary sinus cysts, over 2 test sets	No
Lamassoure et al., 2021 [3]	531 mallet impacts from osteotomies on anatomic models	N/A	COM (impacts kinetics from a receiver in the surgical mallet)	BIN (identification of fractured state of bone after impact)	SVM	ML	Evaluating the state of bone after impact in osteotomies	ACC 0.83, 0.91, and 0.93 with a tolerance of 0, 1, and 2 impacts, respectively	No
Laura et al., 2019 [30]	513 CT scan slices	17:3T:V	E2D (cropped slices from CT scans)	CAT (sinuses and nasal cavity identification)	Darknet-19 deep neural network combined with the You Only Look Once method (YOLO)	DL	Identifying paranasal sinuses and nasal cavities in CT scans	Variable precision and recall rates according to sensitivity of evaluation methods and specific structure, reported graphically	No
Lötsch et al., 2021 [31]	90 patients	N/A	COM (37 nasal anatomy and pathology, olfactory function, quality of life, sociodemographic and clinical parameters)	CON (influence of each criterion on outcomes of ESS)	RF, KNN, SVM, and binary logistic regression	DL	Identifying factors contributing to outcomes of ESS	N/A (weight and role of different features are reported, but the overall model it's not tested)	Built-in feature in freeware software (R)
Murata et al., 2019 [32]	6000 ortopantomographs regions of interest	7:1T:T	E2D (regions of interest from single sinuses on ortopantomographs)	BIN (detection of inflammation)	CNN	DL	Diagnosing maxillary sinus inflammatory conditions on orthopantomographs	ACC 0.875, SEN 0.867, SPE 0.883, AUC 0.875	Open source framework available
Neves et al., 2021 [33]	150 CT scans	13:2T:T	NVI (whole CT scans for testing, manually segmented CT scans for traning)	CON (internal carotid artery, optic nerve and sella turcica auto-segmentation)	Clara SDK-based AHNet7 algorithm	DL	Autosegmenting internal carotid artery, optic nerve, and sella turcica	DSC 0.76 ± 0.12 for the internal carotid artery, 0.81 ± 0.10 for optic nerve, and 0.84 ± 0.08 for sella turcica	Commercial code
Parmar et al., 2020 [34]	447 images	3.5:1T:T	E2D (cropped single side single images from CT scans)	BIN (presence of concha bullosa)	CNN	DL	Identifying conchae bullosae	ACC 0.81 (95% CI 0.73–0.89), AUC 0.93	Open source framework available
Parsel et al., 2021 [35]	545 patients	N/A	COM (22 items from demographic, quality of life, and clinical data)	CAT (association with one of 7 specific clusters associated with specific rhinological diagnoses)	Non-hierarchical cluster analysis performed with partitioning around medoids method	AI	Clustering patients for diagnosis and disease behavior according to their baseline characteristics	N/A (weight and role of different features are reported, but the overall model it's not tested)	Commercial code
Putri et al., 2021 [36]	200 patients	Variable T:T splits	COM (gender, age, air cavity, and Hounsfield unit in CT scan)	BIN (diagnosis of acute sinusitis)	SVM	DL	Identifying patients with maxillary sinusitis from compiled data	ACC up to 1.00	No
Quinn et al., 2015 [37]	331 and 262 regions of interest from 2 cohorts of patients	N/A	COM (autoregressive models from optical flow in regions of interest in videos from optical microscopy from NCS)	CAT (Type of ciliary movement alteration, if any)	SVM	DL	Identifying anomalies in ciliary movement	Best ACC 0.938 and 0.867 for the two tested cohorts	Yes, upon request; open source license software used
Ramkumar et al., 2017 [38]	46 MRIs	3:1T:T	COM (texture analysis from regions of interest in MRI)	BIN (distinguishing inverted papillomas from squamocellular carcinomas)	Diagonal Linear Discriminate Analysis, SVM, and Diagonal Quadratic Discriminate Analysis	ML	Distinguishing between SCC and IP	ACC 0.909 in training and 0.846 in testing for SVM, 0.87% concordance with radiology review	No
Soloviev et al., 2020 [39]	201 optical coherence tomography images	Variable T:T splits	COM (depth-resolved histogram matrix from optical coherence tomography images)	CAT (classification of normal or diseased nasal mucosa, either atrophic or hypertrophic)	KNN, RF, gradient boosting decision trees, support vector clustering, and logistic regression	DL	Classifying normal, atrophic and hypertrophic nasal mucosa from optical coherence tomography images	ACC > 0.94 for all methods for binary classification of normal and pathological tissues; ACC > 0.91 for diagnostic classification of normal, hypertrophic and atrophic tissues	No
Staartjes et al., 2021 [40]	549 images	2:1T:T	N2D (frames from surgical videos)	CAT (identification of septum, inferior turbinate, middle turbinate)	U-Net17 neural network	DL	Identifying anatomical structures in surgical frames from video	36.1% cases correct recognition, 19.2% correct recognition with overshoot, 44.7% incorrect recognition or recognition including 2 or more structures	No
Thorwarth et al., 2021 [41]	80 patients	3:1T:T	COM (peripheral eosinophil count, urinary leukotriene E4 level, and polyp status)	BIN (diagnosis of eosinophil chronic rhinosinusitis)	Artificial neural network	ML	Predicting eosinophilic chronic rhinosinusitis based on preoperative data	AUC of 918 (0.756–0.975) and 0.956 (0.828–0.999) using random and surgeon specific data sets	No
Wirasati et al., 2020 [42]	200 CT scans	7:3T:T	COM (gender, age, air cavity, Hounsfield units)	BIN (diagnosis of acute or chronic rhinosinusitis)	CNN and LSTM	DL	Identifying patients with acute or chronic sinusitis	ACC 0.9833	No
Wu et al., 2020 [43]	26,589 histology slides	14:1:1,3T:V:T	N2D (extracted patches from histology slides)	BIN (diagnosis of eosinophil chronic rhinosinusitis)	CNN	DL	Identifying eosinophil chronic rhinosinusitis on whole histology slides	best AUC 0.974 and 0.957 on validation and testing data sets with InceptionV3	No
Wu et al., 2021 [44]	24,625 patches from histology slides	14:1T:T	E2D (regions of interest from extracted patches from histology slides)	CON (number of eosinophils, lymphocytes, neutrophils, and plasma cells)	CNN	DL	Classifying different subtypes of nasal polyps	Mean absolute errors of the ratios of eosinophils, lymphocytes, neutrophils, and plasma 0.164, 0.213, 0.106, and 0.122%	No

*CT* computed tomography, *NCS* nasal cytology smear, *ESS* endoscopic sinus surgery, *MRI* magnetic resonance imaging; *T:T* train:test, *T:V:T* train:validate:test, *T:V* train:validate, *COM* compiled, *VER* verbal, *N2D* Native 2D graphical, *E2D* elaborated 2D graphical, *NVI* Native volumetric information, *CAT* categorical outcome, *BIN* binary outcome, *CON* continuous outcome, (*AI* artificial intelligence, *DL* deep learning, *KNN* K-nearest neighbors, *SVM* support vector machine, *RF* random forest, *DT* decision tree, *KSPKM* Kernel Spherical K-Means, *S2S* encoder–decoder model, *TRF* transformer model, *HMM* hidden markov model, *LSTM* long–short-term model, *ACC* accuracy, *AUC* area under curve, *CCR* concordance, *CI* confidence interval, *RV* recall value, *SEN* sensitivity, *SPE* specificity, *PPV* positive predictive value, *NPV* negative predictive value, *DSC* Dice similarity coefficient

## Discussion

To the authors’ knowledge, this is the first systematic review addressing the role of AI in rhinology. Our reviews showed that several AI rhinological applications have been developed recently, yet none has been validated in a real-world setting, with a sporadical application of open science principles.

While AI studies often enable claims of boasting efficiency and superiority to human analytical accuracy and speed, their application to real-world scenarios remains far off [45], thus emphasizing the need for an analytical breakdown of articles technical frameworks. Our review revealed that rhinology is not immune to this issue and AI applications remain more theoretical than useful in day-to-day clinics.

Rhinological AI applications appear generally restricted to extremely specific tasks, specifically regulated by the input homogeneity required by AI models and the oversimplifications required to provide answers. Therefore, inputs are often numerically compiled from a prior set of variables. Likewise, graphical information undergoes heavy preprocessing before AI submission. For example, only three reviewed articles used three-dimensional native volume information to allow segmentation of sinonasal structures [25, 27, 33], eight studies used native bidimensional images, and all others used some form of data manipulation.

Theoretically simple analyses such as locating the sinuses in a CT volume remain challenging for AI and only volume estimates have been performed on three-dimensional models. Narrow categorization of answers is required at the output level, therefore, that nearly, half of the reviewed models used dichotomous outputs, while the remaining used predefined categorical answers or continuous numerical scales. This rigid input–output relationship, pivotal for understanding AIs development, is often only hinted at.

The review shows that inconsistent use of reporting parameters hinders an accurate evaluation of rhinological AIs reliability. Reviewed articles employ more than ten different model fitting metrics, the most common being accuracy and area under the curve. As this issue is common to many AI applications, the choice of reporting metrics is a matter of debate among data scientists [45], which led to the development of dedicated metrics, such as *F*_1_ score and Matthews correlation coefficient to replace accuracy, which might be affected by data set imbalances.

The strikingly good performances of reviewed models might nevertheless point toward a potential reporting bias, where less-than-optimal models are not allowed enough editorial space. Publication of negative/intermediate results might allow tackling structural issues and highlight subjects requiring further research or finer model tuning.

Only three studies stated their code was publicly available [12, 23, 37], and few others were adapted from free software or built upon open frameworks. Such source code unavailability hinders testing models on different data sets, thus preventing overfitting which, along with small samples, arbitrary selection of samples, and poor handling of missing data has been exposed as one of the most frequent sources of bias in medical AI studies [46].

Our review further shows that no univocal indications can be drawn for data pool sizes, as published works suggest that models can rely on minimal numbers of patients, though most data sets collected between 10^3^ and 10^4^ items. Analogously train:validation:test splits—required to evaluate algorithm performance with new data—are extremely variable and unrelated to reliability.

Conversely, the choice of AI model appears more consistent. Without consideration of proprietary software, our review shows that the use of CNN for graphical data analysis, SVM for numerical and compiled data analysis, and decision tree/random forest algorithms for making predictions from compiled data represent most scenarios. CNNs are artificial neural networks using a mathematical operation called convolution to fulfill their design task, i.e., process pixel data for image recognition and processing. SVMs are supervised learning models built to analyze data for classification and regression analysis purposes. Although they are able also to handle graphic data, they are not especially designed for this task which is usually addressed with CNNs. Last, decision tree learning is a method commonly used in data mining that aims to create a predictive model of a target variable based on several input variables.

It is also of interest to note how the terms “machine learning” and “deep learning” are used almost interchangeably in the reviewed articles (occasionally in the context of the same article), though they represent different aspects of AI technology. Even if we acknowledge that there is no rigid classification of what constitutes AI, ML or DL, this further supports the notion that there may be a lack of cohesion in AI research. While not intrinsically wrong, such interlabeling hinders the understanding of articles.

There are some limitations to our work that should be considered. In the context of this systematic review, we strived to minimize bias articles selection and data extraction, therefore, not imposing time limits for our searches and including all potential applications. For this purpose, we also decided to include both clinical studies of any design and purely technical studies, though they offer radically different perspectives. While including only articles reporting model reliability minimizes inclusion of purely theoretical studies, it might also have restricted the potential applications presented in this review.

At present, the best AI models available in health sciences are considered non-inferior to expert specialists [47] and are still characterized by technical limits and demands. It comes naturally that rhinology experiences the same distance between AI and everyday practice as other fields of medicine.

## Conclusions

Our review suggests that rhinological AI applications remain only speculative due to the complexities of using data in real-world scenarios. Until more agile algorithms become available on a larger scale, AI will not be able to substitute for clinician work in rhinology. Widespread use of open software policies and lean methodological and technical reporting might allow swifter advances in this field.

## Data Availability

All data pertaining to this systematic review are available from the corresponding author upon reasonable request.
